# Identification of Circovirus Genome in a Chinstrap Penguin (*Pygoscelis antarcticus*) and Adélie Penguin (*Pygoscelis adeliae*) on the Antarctic Peninsula

**DOI:** 10.3390/v12080858

**Published:** 2020-08-06

**Authors:** Hila Levy, Steven R. Fiddaman, Anni Djurhuus, Caitlin E. Black, Simona Kraberger, Adrian L. Smith, Tom Hart, Arvind Varsani

**Affiliations:** 1 Department of Zoology, University of Oxford, South Parks Road, Oxford OX1 3SZ, UK; hila.levy@zoo.ox.ac.uk (H.L.); steven.fiddaman@zoo.ox.ac.uk (S.R.F.); tom.hart@zoo.ox.ac.uk (T.H.); 2 Faculty of Science and Technology, University of the Faroe Islands, Vestarabryggja 15, FO-100 Tórshavn, Faroe Islands; anni.djurhuus@gmail.com; 3 Department of Evolutionary Biology and Environmental Studies, University of Zurich, Winterthurerstrasse 190, 8057 Zurich, Switzerland; caitlin.black@uzh.ch; 4 The Biodesign Center for Fundamental and Applied Microbiomics, Center for Evolution and Medicine, School of Life Sciences, Arizona State University, Tempe, AZ 85287-5001, USA; simona.kraberger@asu.edu; 5 Structural Biology Research Unit, Department of Integrative Biomedical Sciences, University of Cape Town, Observatory, Cape Town 7701, South Africa

**Keywords:** circovirus, penguins, Antarctica, *Pygoscelis antarcticus*, *Pygoscelis adeliae*

## Abstract

Circoviruses infect a variety of animal species and have small (~1.8–2.2 kb) circular single-stranded DNA genomes. Recently a penguin circovirus (PenCV) was identified associated with an Adélie Penguin (*Pygoscelis adeliae*) with feather disorder and in the cloacal swabs of three asymptomatic Adélie Penguins at Cape Crozier, Antarctica. A total of 75 cloacal swab samples obtained from adults and chicks of three species of penguin (genus: *Pygoscelis*) from seven Antarctic breeding colonies (South Shetland Islands and Western Antarctic Peninsula) in the 2015−2016 breeding season were screened for PenCV. We identified new variants of PenCV in one Adélie Penguin and one Chinstrap Penguin (*Pygoscelis antarcticus*) from Port Charcot, Booth Island, Western Antarctic Peninsula, a site home to all three species of Pygoscelid penguins. These two PenCV genomes (length of 1986 nucleotides) share > 99% genome-wide nucleotide identity with each other and share ~87% genome-wide nucleotide identity with the PenCV sequences described from Adélie Penguins at Cape Crozier ~4400 km away in East Antarctica. We did not find any evidence of recombination among PenCV sequences. This is the first report of PenCV in Chinstrap Penguins and the first detection outside of Ross Island, East Antarctica. Given the limited knowledge on Antarctic animal viral diversity, future samples from Antarctic wildlife should be screened for these and other viruses to determine the prevalence and potential impact of viral infections.

## 1. Introduction

Growing interest and access to the Antarctic continent has increased human-induced challenges to Antarctic wildlife while expanding our knowledge of the region’s endemic biota. However, our knowledge of pathogen presence and prevalence in even the continent’s most iconic group of animals, penguins (order: Sphenisciformes), remains limited. To date, most studies of Antarctic penguin viral pathogens have focused on colonies located near fixed scientific research stations, which can monitor sites seasonally or, in some cases, year-round over several years [1,2,3,4,5,6,7,8].

Literature concerning penguin disease primarily consists of observations and case reports of pathologies or mass mortality events in these well-studied colonies. Exploratory studies to determine sub-clinical prevalence of viral pathogens are more recent, progressing from immunoassays, histopathology, plate culture, and microscopic techniques [9,10,11,12,13,14,15,16] to PCR- and sequencing-based methods to survey host viromes and identify particular viruses [4,7,17,18,19,20,21,22]. Though these methodological advances have broadened our knowledge of the viral profile present in the Antarctic environment, more analyses are required to document new viruses and understand viral prevalence.

A recent study of one of the world’s largest penguin colonies, the Cape Crozier Adélie Penguin (*Pygoscelis adeliae*) colony on Ross Island in East Antarctica, identified a novel circovirus associated with Adélie Penguins [3]. Circoviruses (family: *Circoviridae*; genus: *Circovirus*) are circular single-stranded DNA viruses that infect a variety of avian, fish, mammalian, and reptilian species. Their genome encodes two genes in an ambisense orientation: the replication-associated protein (*rep*) in the virion sense and the capsid protein (*cp*) in the complementary sense [23]. The penguin circovirus (PenCV) was first noted in the guano of a chick exhibiting feather disorder in the 2018–2019 breeding season [3]. Subsequent analysis of cloacal swabs from the 2014–2015 breeding season also identified three other individuals with this virus sharing > 99% shared genome-wide identity [3]. Feather disorder in penguins was first observed in an Emperor Penguin (*Aptenodytes forsteri*) chick at Cape Washington, near the Ross Sea in 1996 [3] with subsequent observations in African Penguin (*Spheniscus demersus*) and Magellanic Penguin (*Spheniscus magellanicus*) chicks (Kane et al., 2010). Adélie Penguin chicks with the disorder have been reported three times in the literature: (1) at Esperanza Bay (Hope Bay), Antarctic Peninsula in 2013–2014 [24], (2) at all three colonies on Ross Island in 2011–2012 [2], and (3) the recent case at Cape Crozier, Ross Island in 2018–2019 [3]. A number of viruses have been found to infect Adélie Penguins (reviewed in Smeele et al. [6]), though the etiological agent of the feather disorder has not been confirmed. The recent identification of the PenCV circovirus in an affected individual is intriguing.

Chinstrap Penguins (*Pygoscelis antarcticus*) are closely related to Adélie Penguins (estimated divergence 3.47 mya, 95% Highest Posterior Density: 1.68–5.27) [25]. The southernmost portions of the Chinstrap Penguin range overlap with the northernmost reaches of the Adélie Penguin in the South Sandwich Islands, the South Orkney Island, South Shetland Islands, and down the Western Antarctic Peninsula to 64°S. Where the species overlap, colonies tend to be either entirely single-species or have species-segregated aggregations within the larger colonies. Among the larger, mixed colonies, it is rare to have more than two penguin species breeding. One interesting exception is the Port Charcot colony on Booth Island, Western Antarctic Peninsula, which is home to all three species in the genus *Pygoscelis* [26]. Predominated by the Gentoo Penguin (*Pygoscelis papua*), with approximately 1300–1800 nests, it is also home to 4–10 nests of Adélie Penguins and 16–25 nests of Chinstrap Penguins [27]. The three species nest in very close proximity to each other, which make it a site of interest in exploring host–pathogen interactions in sympatric species. In this paper, we report the molecular detection of sequences of Adélie Penguin circovirus (PenCV) in both an Adélie Penguin and Chinstrap Penguin at Port Charcot, representing the first detection of this virus in the Antarctic Peninsula, 4400 km away from its first reported location at Cape Crozier and the first detection in a Chinstrap Penguin.

## 2. Materials and Methods

**Figure 1:**
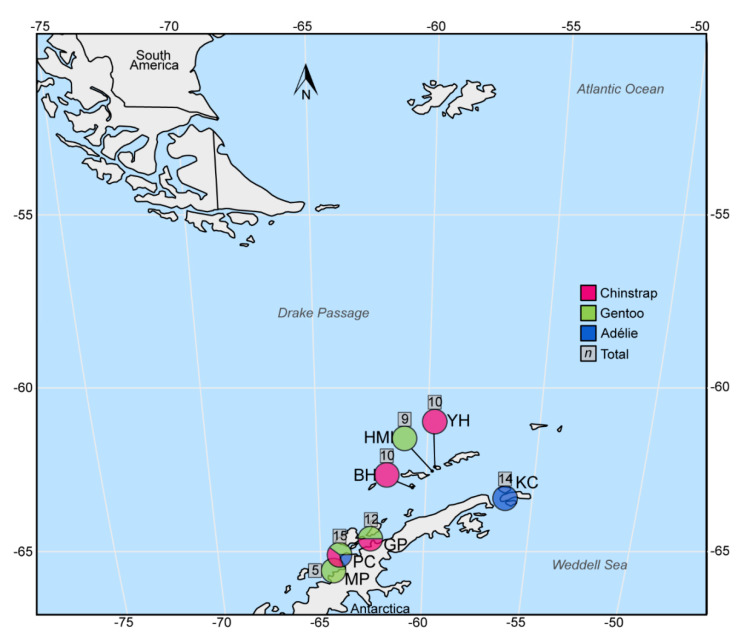
Map of *Pygoscelis* species penguin colonies sampled along the South Shetland Islands and Western Antarctic Peninsula, with pie chart colors representing the species sampled at each site. The grey boxed number associated with each pie chart reflects the total number of swabs obtained at the site. Site abbreviations: Half Moon Island (HMI); Yankee Harbor (YH); Baily Head (BH); Kinnes Cove (KC); Georges Point (GP); Port Charcot (PC); Moot Point (MP) as in Table 1.

Between the 27 December 2015 and the 27 January 2016, a total of 75 cloacal swab samples were obtained from penguins in the genus *Pygoscelis* at seven sites in the South Shetland Islands and the Antarctic Peninsula (Figure 1) as part of a DNA virome study. These consisted of 17 Adélie Penguins (9 adults, 8 chicks), 32 Chinstrap Penguins (21 adults, 11 chicks), and 26 Gentoo Penguins (16 adults, 10 chicks). Regular flocked swabs (Eswab™, Copan^®^, Murrieta, CA, USA) were used, stored in 1 mL of liquid Amies medium, which was stored frozen aside from transport with cold packs under relevant import permits to the United Kingdom. Adult birds were swabbed at the nest when their cloacas were positioned accessibly (facing out), such that the sample could be obtained in under 30 s with minimal disturbance or restraint. In cases of adult–chick paired sampling, the adult was swabbed first, and then chicks were retrieved by hand from the nest, swabbed, and immediately returned to the nest in under one minute. All sampling in Antarctica was conducted under permits S3 34/2015 and S7/S9 35-2015, with ethical approval from the University of Oxford Animal Welfare and Ethical Review Board.

Viral DNA was extracted using 200 µL of the swab suspension after vortexing, using the High Pure Viral Nucleic Acid Kit (Roche Diagnostics, Indianapolis, IN, USA). The virus was preferentially amplified for circular DNA by rolling circle amplification (RCA) using the TempliPhi™ 100 Amplification Kit (GE Healthcare, Chicago, IL, USA). The resulting RCA-amplified DNA (5 µL) was pooled based on animal species per site and used to generate Illumina sequencing libraries using the Nextera DNA Flex Library Prep Kit (Illumina Inc, San Diego, CA, USA). The libraries were sequenced on an Illumina4000 sequencer (2 × 100 bp library), and the resulting paired-end raw reads were trimmed using Trimmomatic [28] and then de novo assembled using metaSPAdes v 3.12.0 [29]. Assembled contigs > 500 nts were analyzed against a viral RefSeq [30] protein database using BLASTx [31]. We identified two contigs (1508 and 1859 nts) in two libraries from Booth Island (one each for Adélie and Chinstrap Penguins) that had high similarity (>85%) to the recently identified penguin circovirus [3]. Based on the sequences of the contigs, we designed a pair of abutting primers (5′-TGAAAGCATGGAGAACTCTGTATAATAAAGT-3′ and 5′-GCGTAATCATTTAATTCGTTCTCGTCATCT-3′) to screen all the individual samples (*n* = 75) and recover the full genomes. The genomes were recovered with this primer pair by PCR using 0.5 µL of the RCA product as a template with KAPA HiFi HotStart DNA Polymerase (Kapa Biosystems, Wilmington, MA, USA) in 20 µL reactions. The amplicons were resolved on a 0.7% agarose gel, excised, purified, and cloned into pJET1.2 plasmid (ThermoFisher, Waltham, MA, USA). The recombinant plasmids were Sanger-sequenced by primer walking at Macrogen Inc. (Seoul, Korea) and contigs assembled using Geneious Prime [32].

The genomes of the circoviruses identified in this study were aligned with the four penguin circoviruses (GenBank MN164703–MN164706) and representative genomes of other avian circoviruses using MUSCLE [33]. The alignment was used to infer a maximum-likelihood phylogenetic tree with 1000 bootstrap replicates using PhyML [34] with GTR+I+G nucleotide substitution model (identified as the best fit model using jModelTest [35]). Similarly, the Rep and CP amino acid sequences encoded by the circoviruses identified in this study were aligned with those of the four penguin circoviruses and representative avian circoviruses. The resulting alignments were used to infer maximum-likelihood phylogenetic trees with 1000 bootstrap replicates using PhyML [34] using the substitution models rtREV+G+I+F for CP and WAG+G for Rep determined as best fit models using ProtTest [36]. For all phylogenetic trees, branches with < 60% branch support were collapsed using TreeGraph2 [37], and the trees were rooted with circoviruses sequences of duck circovirus (DuCV), goose circovirus (GoCV), and swan circovirus (SwCV).

All pairwise identities (genomes and amino acids) were determined using SDT v1.2 [38]. We aligned the PenCV sequences (two from this study and four reported by [3]) and analyzed these for recombination using RDP4 v.4.97 [39].

In order to test for signatures of positive selection, the codeml program in PAML v4.9 [40] was used. Two sets of model comparisons were used: (i) the M1/M2a comparison, where positive selection is disallowed and allowed, respectively, and (ii) the M7/M8 comparison, where dN/dS is modeled using a beta distribution, and positive selection is disallowed and allowed, respectively. The Bayes Empirical Bayes (BEB) algorithm was then used to identify particular sites under positive selection [41].

## 3. Results and Discussion

Of 75 swabs tested in this study, two from Port Charcot, Booth Island, Southwest Antarctic Peninsula, presented penguin circovirus (PenCV) sequences, found in one Adélie Penguin adult (1/2 sampled at the site) and one Chinstrap Penguin adult (1/3 sampled) (GenBank accession numbers MN877414–MN877415). These two circoviruses share 87.1–87.8% genome identity with the ones identified in Adélie Penguins at Cape Crozier (East Antarctica) [3], and since the circovirus species delineation threshold is 80% genome-wide pairwise identity [42], this is considered to be the same species of circovirus. No other sites sampled and no chicks or Gentoo Penguins at this site yielded any circovirus sequences, though this absence may not be indicative of true prevalence due to the small sample size in this study (Table 1).

Though the Booth Island sample size is small, there were only 6 Adélie Penguin chicks and 25 Chinstrap Penguin chicks counted at this colony that year [27], and so this sample set may be considered representative. Gentoo Penguins, on the other hand, had 1023 chicks that year at the site [27], and further sampling would be needed to determine whether this virus might be shared in that species. The absence of virus in the chicks of infected adults could indicate that infection occurs during the non-breeding season elsewhere and/or that this virus is not vertically transmitted or was undetectable in our samples. It is impossible to understand whether this is endemic or an introduction.

**Figure 2:**
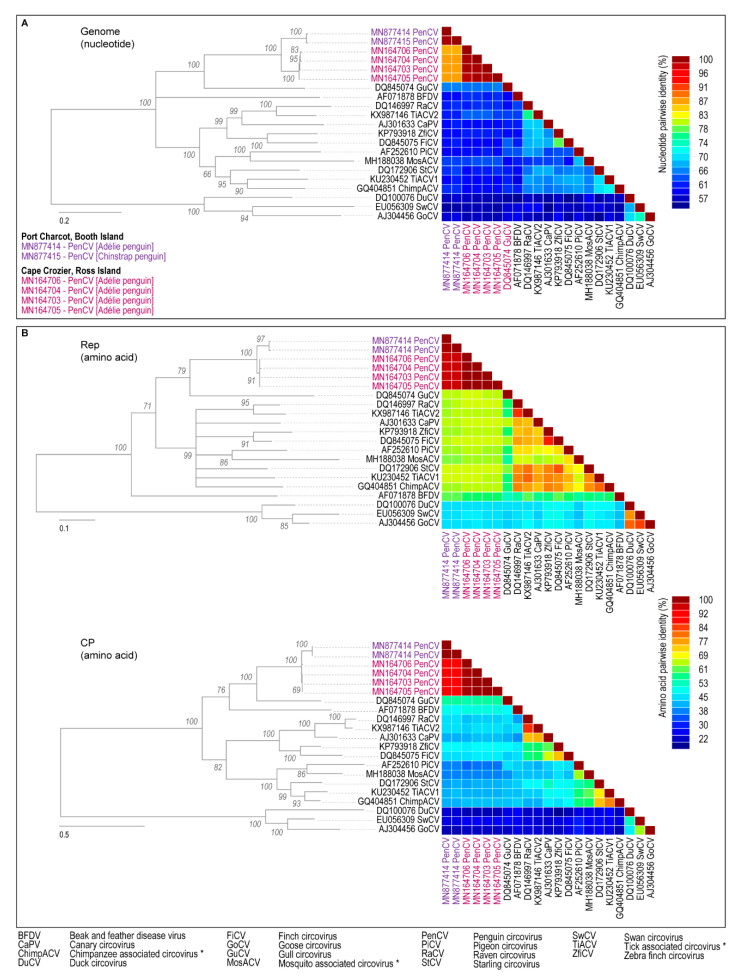
(**A**) Maximum-likelihood phylogenetic tree based on the genomic sequences of PenCVs and other avian circoviruses with a color-coded nucleotide pairwise identity matrix. (**B**). Maximum-likelihood phylogenetic trees of the Rep and CP amino acid sequences encoded by PenCVs with corresponding color-coded amino acid pairwise identity matrixes. Branches with < 60% support have been collapsed, and the trees are rooted with sequences of duck circovirus (DuCV), goose circovirus (GoCV), and swan circovirus (SwCV). * Viruses likely infect avian species, but they have been identified in fecal samples and insect vectors.

Both genomes recovered from our samples were 1986 nucleotides in length, with capsid protein (*cp*; 726 nt) and replication-associated protein (*rep*; 870 nt) genes matching the reported length of PenCV genes. The two sequences share 99.6% genome-wide nucleotide identity and 99.6% (CP) and 99.3% (Rep) amino acid sequence identity with each other, encompassing just two non-synonymous changes in each protein. They share 87.1–87.8% genome-wide nucleotide identity and 90.9–91.7% and 95.8% CP and Rep amino acid identity, respectively, with the PenCV sequences recently discovered ~4400 km away, at Cape Crozier, in three Adélie Penguin adults and one chick (Figure 2, Supplementary Data 1). The circovirus sequences reported in this manuscript are approximately 12% divergent to those reported in Morandini et al. [3] with all PenCV represented in a monophyletic clade based upon whole-genome or encoded protein-specific analyses (Figure 2; Supplementary Data 1). Interestingly, the PenCV reported in this manuscript sampled from Adélie and Chinstrap Penguin (*Pygoscelis antarcticus*) cloacal swabs are more similar to each other than the PenCV from Adélie Penguins from Cape Crozier on Ross Island. Hence, the sequence differences between Booth Island and Ross Island PenCVs appear to be more related to location than a consequence of host species adaptation. In psittacine birds, different host species have been known to share circoviruses (beak and feather disease virus) with > 98% shared genome-wide identity [44]. There was no evidence of recombination between PenCVs sequenced at different locations. 

As might be expected, the level of shared amino acid identity between different circoviruses sequenced from different hosts was much higher for the Rep than for the CP protein (Figure 2B; Supplementary Data 1). It is interesting to note the very high level of identity within the PenCV sequences from Adélie and Chinstrap Penguins from Booth Island even within the CP (> 99.6% amino acid identity). Within the PenCV clade, PAML analysis of the *rep* gene showed no evidence of positive selection (M1/M2a model, *p* = 0.868; M7/M8 model *p* = 0.462; Supplementary Data 2). In contrast, *cp* tended towards significance for the M7/M8 comparison (*p* = 0.102), and one codon was determined to have undergone positive selection using the BEB algorithm (site 201, posterior probability 0.973; Supplementary Data 2). Further increasing the power of this analysis with more penguin circovirus representatives may give a clearer indication of the sites that may be functionally important for endonuclease or helicase function in case of the Rep or receptor bind in the case of the CP. 

## 4. Conclusions

The addition of these new PenCV sequences continues to support their distinct categorization as a separate species from other known avian circoviruses, clustering most closely with gull circoviruses (GuCV) from *Larus* spp., with ~67% genome-wide nucleotide identity. There are no known circovirus sequences identified in other penguin species or seabirds in the next-closest related order Procellariformes. Similar clustering is seen for amino acid sequence-based phylogenies, with PenCVs sharing 66–67% Rep amino acid identity and 57–58% CP amino acid identity with GuCV. PenCV clusters with other avian circoviruses identified in parrot (beak and feather disease virus, BFDV), raven (RaCV), canary (CaCV), zebra finch (ZfiCV), finch (FiCV), pigeon (PiCV), and starling (StCV), sharing 62–64% amino acid identity, with their Reps and CPs sharing 61–68% and 39–53% amino acid identity, respectively.

At the time of sampling, there was no evidence of physical deformities or feather anomalies; therefore, here we report the identification of a new PenCV variant that is not linked to any clinical presentation. Throughout the sampling season (2015–2016), visits to approximately 100 different Pygoscelid penguin colonies in the South Shetland Islands and Antarctic Peninsula yielded no observations of feather disorder, although one was noted in a Rockhopper Penguin in the Falkland Islands. Previous sequencing of PenCV in a chick exhibiting feather disorder in Cape Crozier merits further exploration since feather abnormalities and immunosuppression in other orders of birds—Psittaciformes, Passeriformes, Anseriformes, Columbiformes, Struthioniformes, and Charadriiformes—have been correlated to avian circovirus infections [45]. 

In conclusion, the investigation of prevalence of circoviruses and other potential viral pathogens should be expanded across the range of all penguin species, particularly given their vulnerability to environmental stressors and the rapid pace of abiotic changes to their habitats [46]. 

## Figures and Tables

**Table 1:** Sampling site details and penguin circovirus (PenCV) viral sequence prevalence in adults and chicks. Colony names and subareas follow the Antarctic Site Inventory naming convention [43].

Colony and Antarctic Site Inventory Subarea	Coordinates	Penguin Species (Individuals Sampled)	PenCV Dentification in Adults Sampled	PenCV Identification in Chicks Sampled
Half Moon Island (HMI), South Shetland Islands	60°36′ S, 59°55′ W	Chinstrap (10)	0/10	-
Yankee Harbor (YH), Greenwich Island, South Shetland Islands	62°32′ S, 59°47′ W	Gentoo (9)	0/6	0/3
Baily Head (BH), Deception Island, South Shetland Islands	62°58′ S, 60°30′ W	Chinstrap (10)	0/5	0/5
Kinnes Cove (KC, Madder Cliff), Joinville Island, Northeast Antarctic Peninsula	63°18′ S, 56°29′ W	Adélie (14)	0/7	0/7
Georges Point (GP), Ronge Island, Northwest Antarctic Peninsula	64°40′ S, 62°40′ W	Chinstrap (6)	0/3	0/3
		Gentoo (6)	0/3	0/3
Port Charcot (PC), Booth Island, Southwest Antarctic Peninsula	65°05′ S, 64°00′ W	Adélie (3)	1/2	0/1
		Chinstrap (6)	1/3	0/3
		Gentoo (6)	0/3	0/3
Moot Point (MP), Southwest Antarctic Peninsula	65°12′ S, 64°06′ W	Gentoo (5)	0/4	0/1
Total by Species		Adélie (17)	1/9	0/8
		Chinstrap (32)	1/21	0/11
		Gentoo (26)	0/16	0/10

## Supplementary Materials

The following are available online at https://www.mdpi.com/1999-4915/12/8/858/s1, Supplementary Data 1: Spreadsheet containing pairwise identities of the avian circovirus genomes, and the Rep and CP amino acid sequences; Supplementary Data 2: Bayes Empirical Bayes inference of the probability of positively selected sites in PenCV protein-coding genes obtained from various codeml models [41].
