# Supplementary material for: Identification of Circovirus Genome in a Chinstrap Penguin (Pygoscelis antarcticus) and Adélie Penguin (Pygoscelis adeliae) on the Antarctic Peninsula
Source: Viruses. 2020 Aug 6;12(8):858. doi: 10.3390/v12080858 (PMC7472332; doi:10.3390/v12080858)
Supplement: Supplementary file 1 [file viruses-12-00858-s001.zip › Supplementary Data 2.docx]

| **Gene** | **Model** | **Log-likelihood** | **2 x (ΔlnL)** | **Chi^2^** | **Selected sites (Pr)** |
| --- | --- | --- | --- | --- | --- |
| *cp* | M1 | -1206.358425 | 3.2881 | 0.193 | 177 (0.640); 201 (0.934) |
| *cp* | M2a | -1204.714375 |  |  |  |
| *cp* | M7 | -1207.004397 | 4.573534 | 0.102 | 177 (0.733); 201 (**0.973**) |
| *cp* | M8 | -1204.717630 |  |  |  |
| *rep* | M1 | -1609.231633 | 0.284004 | 0.868 | 33 (0.755) |
| *rep* | M2a | -1609.089631 |  |  |  |
| *rep* | M7 | -1609.865526 | 1.545972 | 0.462 | 33 (0.887) |
| *rep* | M8 | -1609.092540 |  |  |  |
